# Characterisation and internalisation of recombinant humanised HMFG-1 antibodies against MUC1

**DOI:** 10.1038/sj.bjc.6602847

**Published:** 2005-11-01

**Authors:** L M Pericleous, J Richards, A A Epenetos, N Courtenay-Luck, M P Deonarain

**Affiliations:** 1 Department of Biological Sciences, Imperial College London, Exhibition Road, London SW7 2AZ, UK; 2 Antisoma Research Laboratories, West Africa House, Hanger Lane, London W5 3QR, UK

**Keywords:** immunoglobulin, Fab fragment, affinity, mucin, internalisation

## Abstract

The humanised HMFG-1 immunoglobulin has been extensively developed as a clinical immunotherapeutic agent for MUC1 expressing tumours. We have constructed a single-chain Fv (scFv) and Fab fragment from this antibody and shown that both these species retain their specificity for MUC1. The scFv was less stable and less soluble than the Fab. Detailed analyses of the binding kinetics of the whole IgG and Fab fragment show that the affinity for MUC1 synthetic peptides is low (approximately 100 nM for the IgG and 10 *μ*M for the Fab), with particularly low but similar dissociation rate constants (0.031–0.095 s^−1^). Binding to native antigen on the cell surface is over two orders of magnitude better. Confocal immunofluorescence microscopy shows that both the IgG and Fab are internalised rapidly (the IgG is internalised within 15 min) and colocalise to early endosomes. This work provides an appreciation of the binding, internalising and trafficking kinetics, important for the development of future therapeutics based on this antibody.

Mucins are a family of glycoproteins expressed on the apical surface of most epithelial cells. Their structural composition offers cell surface protection and the homeostasis of the local molecular microenvironment. Transmembrane mucins may function as cell surface receptors conducting signals leading to responses such as proliferation or apoptosis, whereas secreted mucins are predominantly implicated as a first line of defence for the epithelial surface (reviewed in Hollingsworth and Swanson, 2004).

Mucins are comparatively large glycoproteins with molecular weights of over 1000 kDa and a biochemical composition of 50–80% O-linked oligosaccharides. The major features of mucins are the tandem repeat domains, sets of repeated amino-acid sequences rich in serine, threonine and proline. In the case of MUC1, each repeat unit typically consists of between 20 and 30 amino acids, with up to 500 repeats per polymorphic mucin molecule (Gendler *et al*, 1990).

The most well characterised mucin is MUC1 a type 1 transmembrane protein with the 20 amino-acid tandem repeat sequence ‘PDTRPAPGSTAPPAHGVTSA’ (Burchell *et al*, 1989), an SEA domain (which associates with lipid rafts) and a short C-terminal cytoplasmic tail of 72 amino acids. The cytoplasmic tail, which is highly conserved between species, contains seven tyrosine residues, four of which lie in signalling pathway motifs (Zrihan-Licht *et al*, 1994). Chimeric receptor studies have identified which of these tyrosine residues are phosphorylated upon ligand binding (Wang *et al*, 2003), but the exact nature of the true ligand is as yet unknown. The cytoplasmic tail has also been shown to bind and signal through *β*-catenin and the mitogen-activated kinase pathways (Yamamoto *et al*, 1997; Schroeder *et al*, 2001). MUC1 is proteolytically cleaved upon post-translational processing forming two associated subunits at the cell surface (Parry *et al*, 2001).

For many years mucins have been implicated in the pathogenesis of cancer, especially adenocarcinomas (Apostolopoulos *et al*, 1999; Hu and Xing, 2003; Hollingsworth and Swanson, 2004). It is postulated that tumour cells utilise mucins to remodel the local microenvironment to promote tumour invasion, metastasis and growth (Hollingsworth and Swanson, 2004). Overexpression of MUC1 and the expression of aberrant forms of MUC1, typified by the loss of apical distribution, have been reported in many carcinomas (Zotter *et al*, 1988), in particular breast and ovarian (Apostolopoulos *et al*, 1999; Hu and Xing, 2003). This aberrant expression is a consequence of either the deregulation of the *MUC1* gene itself or the genes encoding the enzymes, which modify the MUC1 protein. Clatherin-mediated endocytosis of MUC1 is stimulated two-fold and its intracellular trafficking and subcellular distribution is altered as a probable consequence of the aberrant glycosylation (Altschuler *et al*, 2000).

This differential glycolsylation allows MUC1 to be exploited as a pan-carcinoma tumour-associated antigen with many B-cell (Burchell *et al*, 1983) and T-cell (Jerome *et al*, 1991) epitopes. To date, more than 50 monoclonal antibodies specific for the MUC1 protein have been described (Price *et al*, 1998). Most recognise exposed epitopes within the 20-mer tandem repeat region accessible due only to the differential glycosylation, thereby affording these antibodies a degree of specificity for the cancer cell. The most commonly recognised epitope is the hydrophilic ‘PDTRPAP’ sequence (Petrakou *et al*, 1998), which is predicted to have a relatively unstructured conformation with a simple ‘fist-like’ projection (Price *et al*, 1998).

The MUC1 specific murine monoclonal antibody HMFG-1 was raised against human milk fat globules, a rich source of MUC1 and was subsequently shown to bind the exposed tetra-peptide sequence ‘PDTR’ (Taylor-Papadimitriou *et al*, 1981). HMFG-1 and a humanised version of this antibody HuHMFG-1, which has demonstrated similar specificity and affinity for MUC1 (Verhoeyen *et al*, 1993), have both been evaluated in clinical trials. Iodine-123-labelled HMFG-1 was successfully used to detect MUC1 positive tumours in patients with primary and metastatic lesions of ovarian, breast and gastrointestinal cancer (Epenetos *et al*, 1982; Al-Yasi *et al*, 2002). A phase I/II clinical trial using yttrium-90 conjugated murine HMFG-1 (R1549) showed promise in patients with ovarian cancer (Hird *et al*, 1993). A follow-up phase III ‘SMART’ trial, however, concluded that the antibody when given after standard chemotherapy did not result in an increased survival benefit compared to standard therapy alone (Seiden and Benigno, 2004). The unmodified humanised antibody (R1550) is currently being developed as an immunotherapeutic for breast cancer (Courtenay-Luck N, pers. comm.; http://www.antisoma.com/products/R1550_Therex.asp) supported by evidence that this antibody can elicit a strong antibody directed cellular cytotoxicity response (Snijdewint *et al*, 2001).

As yet the potential of MUC1 as a tumour-associated antigen that internalises has not been widely evaluated with respect to the delivery of chemotherapeutic agents or toxins. The route and kinetics of HuHMFG-1 internalisation are unknown and are critical for the successful delivery of cytotoxic agents, which exert their action in intracellular compartments. Addressing these issues we constructed the single-chain Fv (scFv) and Fab recombinant antibody fragments of HuHMFG-1 and assessed stability, affinity and internalisation characteristics alongside the parent antibody. The scFv was found to be insoluble and unstable and required a stabilising mutation (Krauss *et al*, 2004). This is the first report delineating the internalisation pathway of the clinically tested HuHMFG-1 antibody with respect to antigen binding and intracellular trafficking. Furthermore, surface plasmon resonance (SPR)-measured binding data for the Fab and parent antibody provide a fuller insight into the previously uncharacterised binding kinetics and affinity profiles of these antibodies and is discussed in relation to the observed internalisation characteristics.

## MATERIALS AND METHODS

### Materials

Humanised HMFG-1 monoclonal antibody was of clinical grade purity and supplied by Antisoma Research Ltd, UK. Proteolytically derived HuHMFG-1 Fab was prepared using a Pierce Fab preparation kit following the manufacturer's instructions. Anti-HIS antibody was from Qiagen, Crawley, UK, Transferrin-Alexa Fluor 594 was from Molecular Probes (Invitrogen, Paisley, UK). Anti-mouse and anti-human-peroxidase and FITC conjugates were from Sigma, Dorset, UK. pET20b vector and *Escherichia coli* BL21(DE3) pLysE strains were from Novagen, Nottingham. A fusion protein consisting of glutathione-*s*-transferase and one MUC1 tandem repeat was a kind gift from Dr R Verma (Antisoma Research Ltd, UK). The 60-mer MUC1 peptide containing three tandem repeats (Henderikx *et al*, 2002) was a kind gift from Dr P Henderikx (Dyax SA, Belgium). Anti-c-myc monoclonal antibody 9E10 was a kind gift from Professor N Lemoine (CRUK, London). Plasmids pAS1 and pAS2 were kind gifts from Dr R Young (Antisoma Research Ltd, UK).

### Cell culture

The human tumour cell lines MCF-7, SKOV-3 and COS-7 were obtained from the European Collection of Cell Cultures (ECACC) and maintained in Eagle's modified essential medium (EMEM) with 10% foetal calf serum (FCS), McCoys medium with 15% FCS and Dulbecco's modified essential medium (DMEM) with 10% FCS respectively. All cells were cultured in 75 cm^2^ flasks and passaged at 70–90% confluency.

### Construction of the HuHMFG-1 Fab expression vector

**Figure 1:**
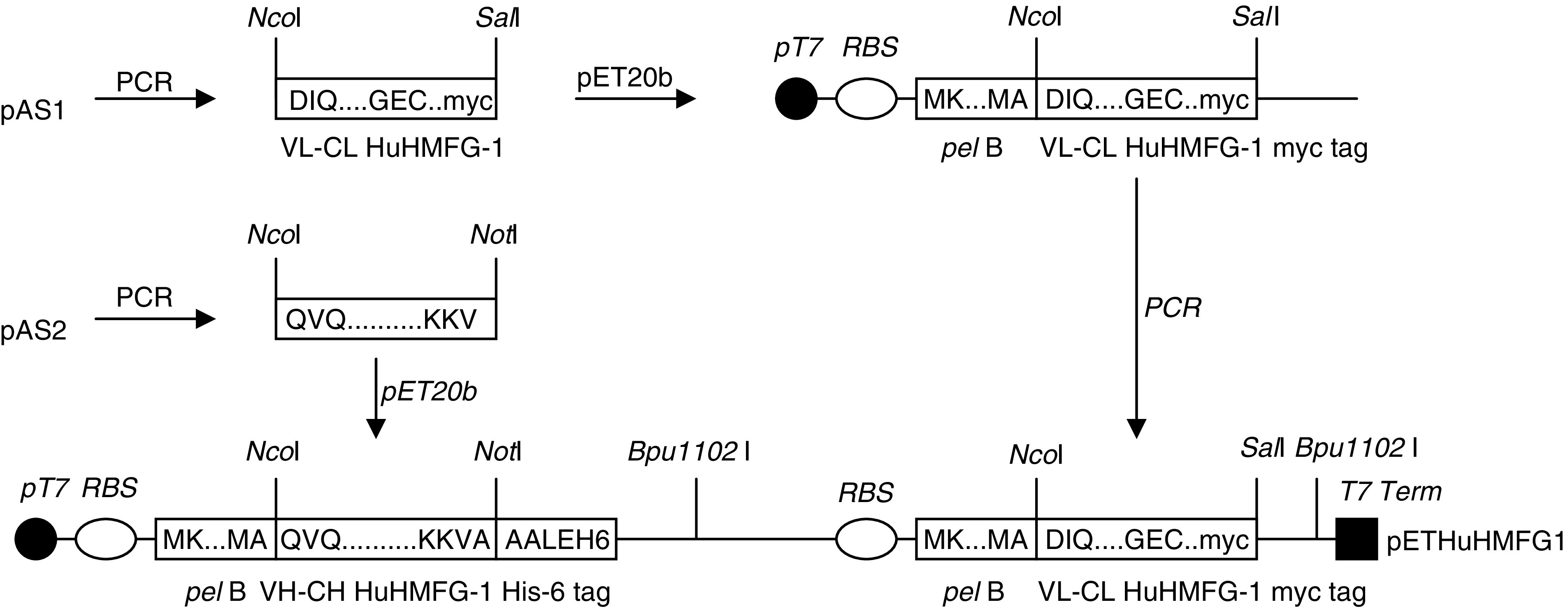
Construction scheme for the HuHMFG-1 Fab. Plasmids pAS1 and pAS2 containing the light chain and heavy chain of the HuHMFG-1 immunoglobulin were used as templates to PCR amplify the fragments for Fab construction. These were cloned into pET20b+ individually and *Nco*I/*Sal*I and *Nco*I/*Not*I subgenes respectively. Then, the whole expression cassette for the light chain was amplified and cloned into the *Bpu*1102I site of the pET vector to form the pETHuHMFG1 expression vector.

The VH-CH1 domain of the HuHMFG-1 Fab was PCR amplified from plasmid pAS2 using the primers VHCH-N (5′ CGCCTCCATGGCAATCGATCAGG TGCAGCTGGGTGCAG 3′) and VHCH-C (5′ GGCGCGCGGCCCGCATCGATAGCAACTTTCTTGTCCA CCTT 3′), and ligated into the *Nco*I and *Not*I restriction sites of pET20b+. This clone was called pET20bHMFG1-H. The VL-CL domain was PCR amplified from pAS1 using the primers VLCL-N (5′ TCC GCCTCCATGGCCGACATCCAGATGACCCAGAGC 3′) and VLCL-C (5′ GGAGGCGTCGACCTAATTC AGATCCTCTTCTGAGATGAGTTTTTGTTCACTCTCTCCCCTGTTGAA 3′) and ligated into the *Nco*I and *Sal*I restriction sites of pET20b+. This clone was called pET20HMFG1-L. The *Bpu*11021I and *Nco*I restriction sites were removed from the CL and VL domains, respectively, by silent site-directed mutagenesis. Following this mutagenesis, the whole expression cassette of pET20bHMFG1-L was PCR amplified using primers VLCL-N2 (5′ GCTCGCTAGCTGAGCAATTTTGTTTAACTTTAAGAAGG 3′) and VLCL-C2 (5′ CGAGCGATGCTCAGCGTCGAGCTAATTCAGATCCTCTTCTGA 3′) and ligated into the *Bpu*11021I restriction site of pET20bHMFG1-H, which is located 3′ to the heavy chain gene. The integrity of the resulting HuHMFG-1 Fab expression vector, called pETHuHMFG1, was confirmed by restriction digest analysis and DNA sequencing. This clone comprised of the Fab heavy chain (VH-CH1) with a C-terminal hexa-histidine tag, the light chain (VL-CL) with a C-terminal c-myc tag, with both chains preceded by a *pel* B secretion signal and a ribosome binding site, and the whole construct under the control of a single T7 promoter (Figure 1).

### Expression and purification of the HuHMFG-1 Fab

**Figure 2:**
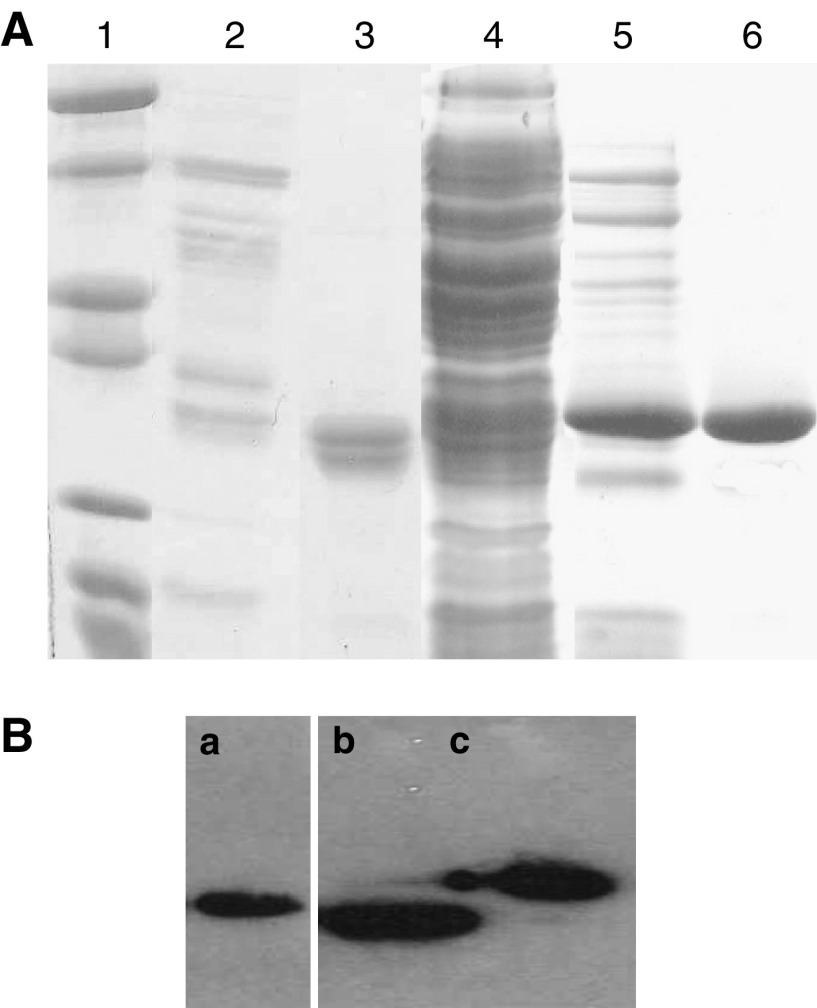
Expression and purification of the HuHMFG-1 Fab and scFv. (**A**) The Fab was isolated by IMAC from concentrated supernatant (lane 2) to yield pure protein with both chains intact (lane 3). The scFv expressed insolubly (lane 4), recovered as insoluble inclusion bodies (lane 5) and purified after refolding (lane 6). Molecular weight markers are shown in lane 1 (116, 66, 45, 35, 25, 18, 14 kDa). (**B**) Anti-c-myc Western blot analysis detects the Fab light chain (a), Anti-His-6 blotting detects the Fab heavy chain (b) and scFv (c). This shows that all the antibody fragments are full-length proteins.

Soluble HuHMFG-1 Fab was expressed in 1 l cultures of *E. coli* BL21 (DE3) pLysE (Studier, 1991), grown in 2TY media with 100 *μ*g ml^−1^ ampicillin and 25 *μ*g ml^−1^ chloramphenical at 37°C and induced with 0.1 mM IPTG for 16 h at 30°C. The culture was then centrifuged and the clarified supernatant was filtered (0.2 *μ*m), concentrated 10-fold, dialysed into PBS/1 M NaCl and subsequently purified by immobilised metal affinity chromatography (IMAC) on chelated cobalt sepharose (Talon®, Clontech, Oxford, UK). Further purification was achieved by size exclusion chromatography using a Superdex-75 10/300 column equilibrated in PBS. The protein eluted at its predicted native molecular weight of 50 kDa (data not shown) and SDS–PAGE/Western blot analysis indicated that the purified protein contained both Fab chains with the expected molecular weight (Figure 2).

### Construction and expression of the HuHMFG-1 scFv gene

The gene encoding the HuHMFG-1 scFv was constructed by PCR amplification and sequential cloning of the VH and VL antibody domains from the pAS2 and pAS1 vectors, respectively, as previously described (Krauss *et al*, 2004). Bacterial expression resulted predominantly in inclusion body formation. Functional scFv could be obtained from periplasmic preparations (Krauss *et al*, 2004), but higher yields of similarly functional scFv could be obtained from refolding experiments (Deonarain *et al*, 1997) and subsequent IMAC purification yielding pure protein as analysed by SDS–PAGE and Western blot (Figure 2).

### Antigen and fixed cell ELISAs

Micotitre plates (96-well) were either coated overnight with 5 *μ*g ml^−1^ of GST-MUC1 in PBS at 4°C, or seeded with approximately 10^6^ cells/well, grown overnight and fixed with 2% paraformaldehyde for 30 min. Following the addition of 50 *μ*l well^−1^ of serially diluted purified antibody or appropriate control samples in PBS/2% skimmed milk powder (Marvel®), binding was detected using mouse anti-His (scFv or Fab heavy chain) or mouse anti-c-myc (Fab light chain) followed by an anti-mouse peroxidase conjugate. Goat anti-human Fab-specific peroxidase conjugate antibody was also used to detect the binding of the Fab and whole HuHMFG-1 antibody for direct comparisons. Each antibody incubation step was followed by three PBS/0.1% Tween-20 washes and three PBS washes. All ELISAs were developed with *o*-phenyldiamine (OPD) substrate. In the thermal stability studies, antigen ELISAs were carried out after a 10-min incubation in PBS at various temperatures.

### Flow cytometric analyses

SKOV-3 or COS-7 cells were harvested from 75 cm^2^ flasks by washing with sterile PBS and dissociation with Trypsin/EDTA. Cells were washed twice in ice-cold DMEM/2% FCS and 10^6^ cells resuspended in 10–100 *μ*g ml^−1^ of primary antibody in a volume of 0.2 ml for 1 h at 4°C. Binding was detected using either 5 *μ*g ml^−1^ anti-human Fab specific FITC- (HuHMFG1 IgG and Fab) or 5 *μ*g ml^−1^ mouse anti-His FITC- (scFv) conjugates in 0.2 ml for 1 h at 4°C. Each antibody incubation step was followed by the centrifuging (2 min at 600 **g**) and resuspention of the cells three times in ice-cold PBS/2% FCS. Samples were analysed using a 3-colour Becton Dickinson FACSort machine at a single laser excitation wavelength of 460–500 nm. Data were analysed using CellQuest software.

### BIACore SPR binding kinetics

Studies were conducted using a BIACore 3000 (BIACore AB, Upsala, Sweden) with version 4.0 control software. Carboxy-Methyl Dextran (CM5) sensor chips were coated with approximately 1000 or 3000 response units (RU) of GST-MUC1 peptide on the test flow cells, and either approximately 800 or 2400 RU of free GST on the negative control flow cells. Standard amine coupling methodology was used. Streptavidin (SA) sensor chips were coated with approximately 150 RU of nonspecific biotinylated peptide (17-mer) ‘NH_2_-CYWFSRSGKAWADADNY-COOH’ on negative control flow cells and approximately 50 RU of biotinylated MUC1 60-mer peptide ‘NH_2_-(VTSAPDTRPAPGSTA PPAHG)_3_-COOH on test flow cells. Wizard-driven kinetic binding experiments, set at a flow rate of 30 *μ*l min^−1^ to minimise rebinding and mass transfer effects, were run in PBS containing 0.05% Tween-20. Antibody samples serially diluted in running buffer were tested in duplicate with the final data sets collated from two separate experiments. Sensogram data were analysed using the BIA-evaluation software version 3.1 with affinity and avidity binding constants determined using either Langmuir 1 : 1, bivalent or steady-state binding models. Automated ‘global’ curve fitting of data sets typically yielded *χ*^2^ values between 5 and 20.

### Confocal immunofluorescent microscopy

SKOV-3 cells were seeded onto 13 mm coverslips and grown to 80% confluency. The cells were washed twice for 10 min at room temperature with Leibovitz medium (L-15) supplemented with 0.2% BSA and 0.1 mg ml^−1^ iron nitrate (to eliminate any growth factors and excess transferring) and chilled to 4°C. Transferrin-Alexa Fluor 594 and the appropriate HuHMFG-1 antibody (5–100 *μ*g ml^−1^) were applied to the cells at 4°C for 30 min. Three washes with chilled L-15/0.2% BSA/0.1 mg/ml iron nitrate were conducted before incubation at 37°C for various time points, followed by fixation for 15 min in 4% paraformaldehyde. Cells were permeablised and blocked with 0.1% saponin/1% BSA/PBS for 1 h at room temperature before applying 5 *μ*g ml^−1^ anti-human Fab-specific FITC (diluted in 0.1% saponin/1% BSA/PBS) for a further 60 min. This was followed by six 10 min washes with 0.1% saponin/1% BSA/PBS and mounting of the coverslips onto microscope slides, using Vectashield™ (Vectalabs) mounting medium and nail varnish. Slides were viewed on a Zeiss LSM 510 laser-scanning confocal microscope. The LSM 510 software was used for image processing. The Volocity® programme was used to re-construct 3D images and movies from image slices.

## RESULTS

### Production of the HuHMFG-1 scFv and Fab

**Figure 3:**
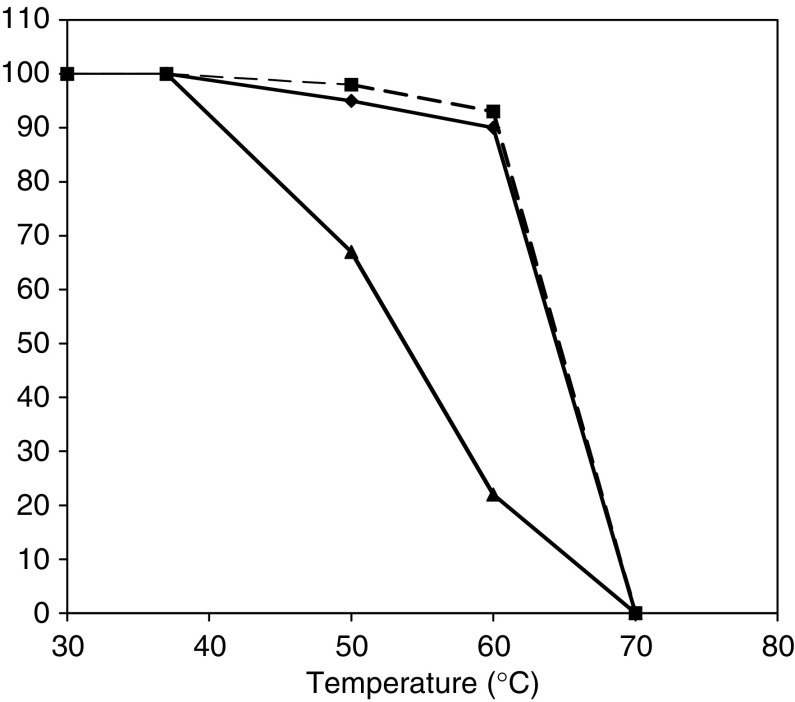
Thermostability profile of HuHMFG-1 antibodies. Antibody samples were incubated at the temperatures indicated and the percentage binding activity (by ELISA) was plotted. IgG (closed squares), Fab (closed diamonds) and scFv (closed triangles). The Fab is seen to be at least 10°C more thermo-stable than the scFv fragment.

The bacterial expression of HuHMFG-1 scFv led predominantly to insoluble inclusion body formation with limited yields of soluble, functional protein obtained following refolding and IMAC purification (Figure 2). In contrast, expression and purification of the HuHMFG-1 Fab fragment yielded soluble protein at 4 mg l^−1^ of bacterial culture (Figure 2). Furthermore, the nonproblematic soluble expression of the individual heavy (VH-CH1) and light (VL-CL) chains from pET20bHuHMFG1-H and pET20bHuHMFG1-L, respectively, suggested that the constant domains were the source of the increased stability and solubility of the Fab in comparison with the scFv (data not shown). Fab stability on storage (data not shown) or after prolonged heat treatment, at a range of temperatures was comparable to the parent IgG (Figure 3). The scFv, however, exhibited significantly poorer stability compared to other antibody species with an 80% decrease in binding at 60°C (Figure 3). Fab antibody was also made from the proteolysis of whole HuHMFG-1 IgG for comparison.

### Cell and antigen binding assays

**Figure 4:**
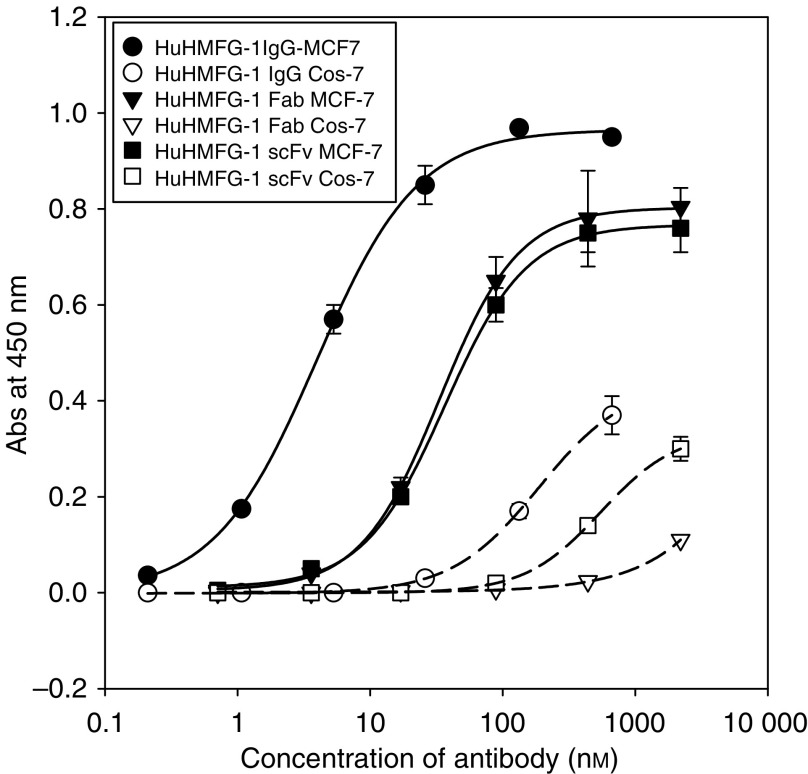
HuHMFG-1 cell ELISAs. Cell ELISAs of the various HuHMFG1 antibodies on antigen-positive cells (MCF-7) and -negative cells (Cos-7). For binding to MCF-7 tumour cells, IgG (closed circles), Fab (closed triangles) and scFv (closed squares). For binding to Cos-7 cells, IgG (open circles), Fab (open squares) and scFv (open triangles). Binding curves were fit to a sigmoidal 4-logistic equation (SigmaPlot) and mid-points of binding (equivalent to the apparent equilibrium dissociation constants, *K*_D_s) determined. Apparent *K*_D_s determined from these are IgG=3.9±0.4 × 10^−9^ M, recombinant Fab=3.34±0.09 × 10^−8^ M, proteolyic Fab=3.16±0.3 × 10^−8^ M (not shown) and scFv=3.65±0.2 × 10^−8^ M.

**Figure 5:**
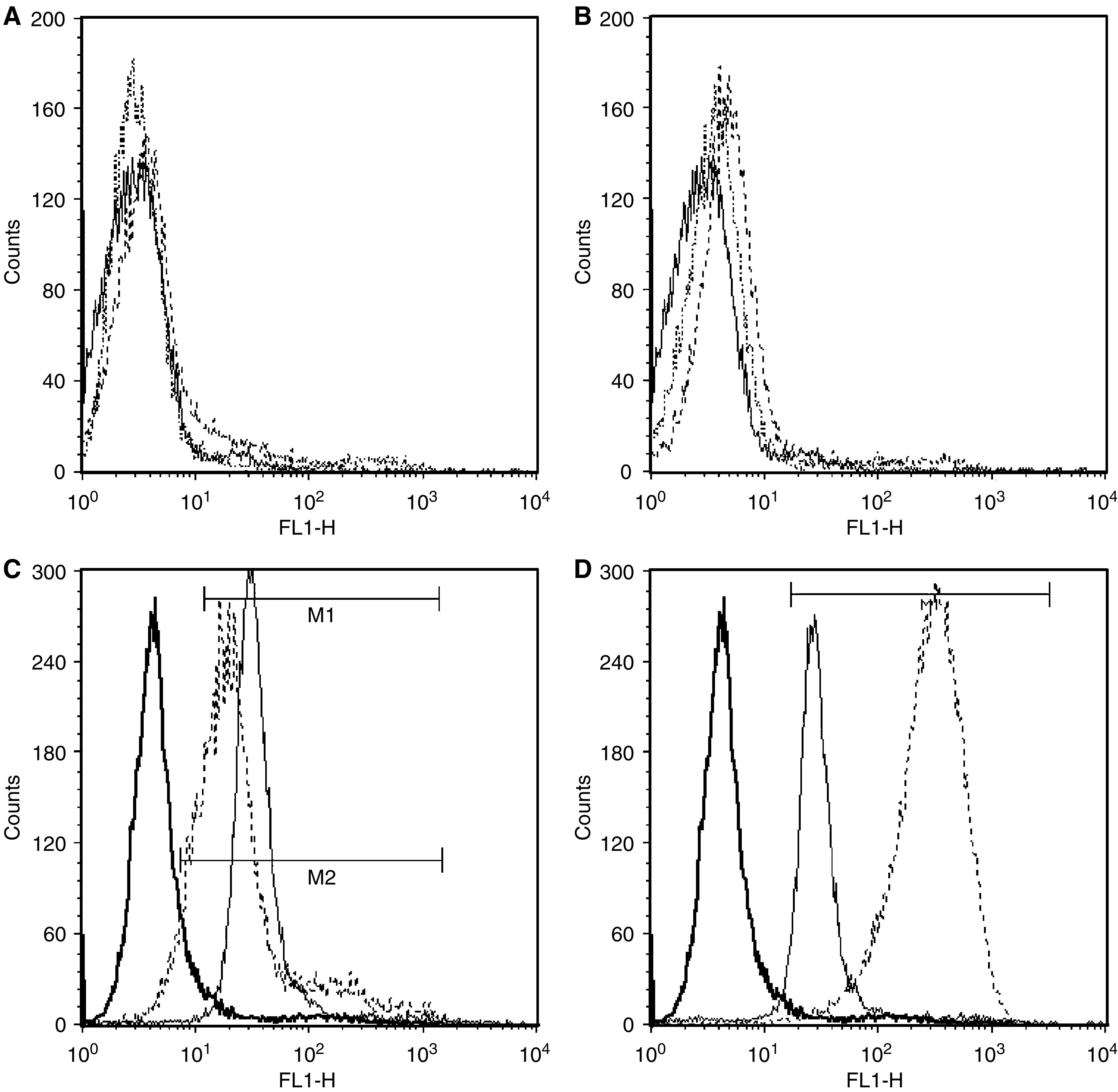
Flow cytometric analyses of live cell binding. (**A** and **B**) Binding to antigen-negative COS-7 cells and (**C** and **D**) binding to antigen-positive SKOV-3 cells. (**A**) Bold line=cells/detection antibodies only, thin line=HuHMFG-1 scFv, dashed line=proteolytically-derived Fab. (**B**) Bold line=cells/detection antibodies only, thin line=recombinant Fab, dashed line=whole HMFG-1 IgG. (**C**) Bold line=cells/detection antibodies only (geometric mean of fluorescence intensity 4.7), thin line=proteolytically-derived Fab (34.1), dashed line=HuHMFG-1 scFv (23.3). (**D**) Bold line=cells/detection antibodies only (4.7), thin line=recombinant Fab (29.5), dashed line=whole HMFG-1 IgG (275.6). The Fab species show similar binding with the whole IgG binding live cells more effectively.

Both the recombinant HuHMFG-1 Fab and scFv showed specific antigen binding by ELISA against the GST-MUC1 peptide, demonstrating retention of the antigen specificity of the parent IgG. Additionally, no significant binding was detected against a range of nonspecific protein antigens, including BSA, lysozyme and unconjugated GST. The binding affinity was low with saturating binding not being achieved (data not shown). Comparative ELISAs against fixed MCF-7 cells (Figure 4), which express the native MUC1 tumour antigen, gave reasonably high apparent affinity values (IgG – 3.9 × 10^−9^ M, recombinant Fab – 3.3 × 10^−8^ M, proteolytic Fab – 3.2 × 10^−8^ M (not shown) and scFv – 3.7 × 10^−8^ M), which were at least 10-fold (and approaching 100-fold) better than the peptide binding for each antibody species. Analysis by flow cytometry using antigen positive (SKOV-3) and antigen negative (COS-7) live cells demonstrated further the specificity of these recombinant antibodies. Significant shifts in fluorescence intensity were observed with the antigen positive cell line only for each of the three antibody species, recombinant scFv, recombinant and proteolytically derived Fab and parent IgG (Figure 5). Compared to the FITC-treated cell control (mean fluorescence=4.49 U), scFv binding resulted in a shift to 23.3 U, whereas the proteolytic Fab resulted in a shift to 34.1 U, recombinant Fab resulted in a shift 29.5 U and the whole IgG resulted in a shift 275.6 U.

### SPR kinetic binding studies

**Figure 6:**
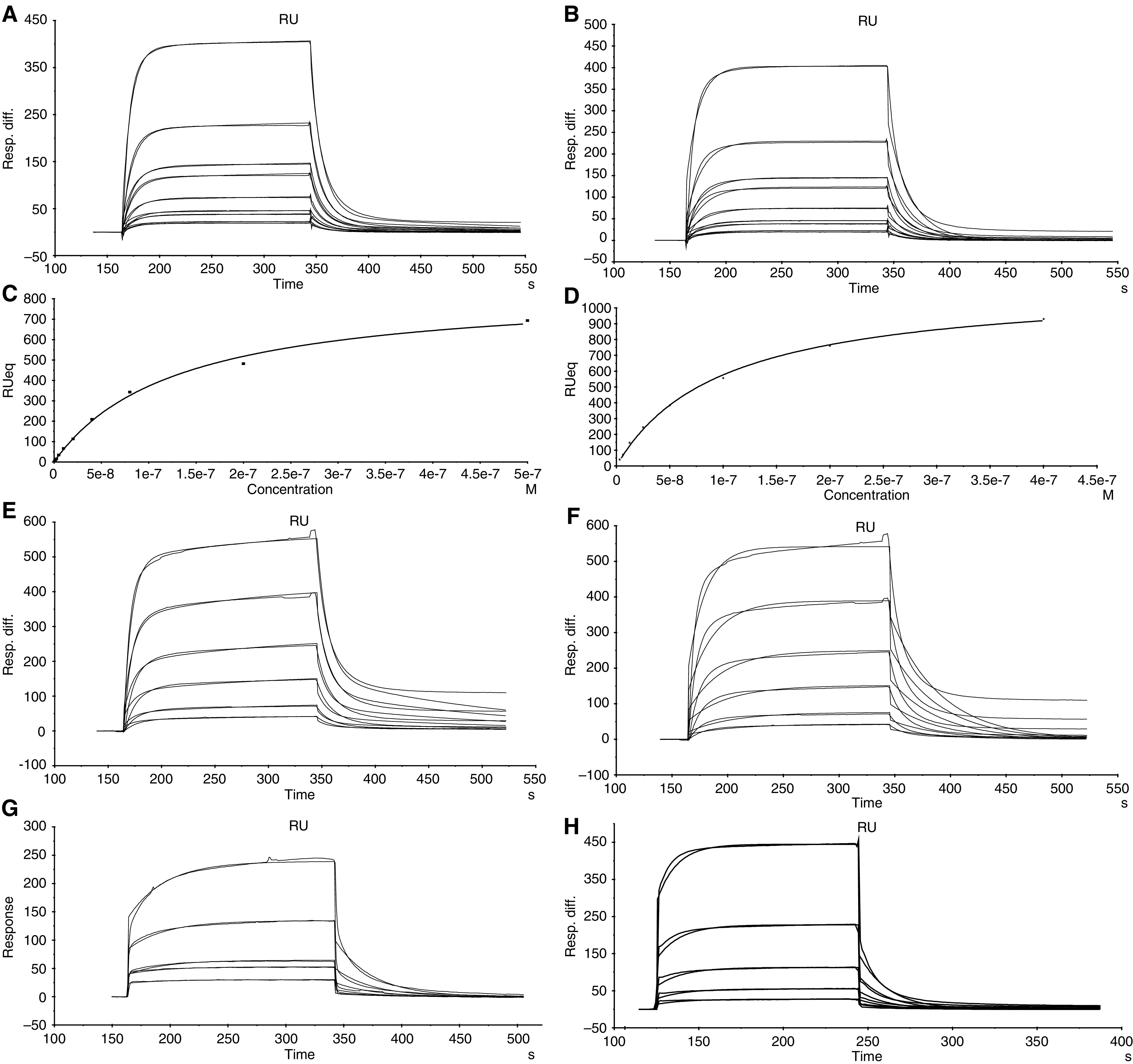
BIAcore surface plasmon resonance kinetic analyses. Sensogram plots for IgG and recombinant Fab binding experiments on immobilised Gst-MUC1 peptide and 60-mer biotinylated peptide. Approximately 3 min of association and 3 min of dissociation are shown. IgG binding curves for the Gst-MUC1 antigen fit to (**A**) bivalent analyte model and (**B**) 1 : 1 binding model. Steady-state binding curves for Gst-MUC1 (**C**) and 60-mer peptide (**D**). IgG binding curves for the 60-mer antigen fit to a bivalent analyte model (**E**) and a 1 : 1 binding model (**F**). Fab binding curves, fit to a 1 : 1 model on Gst-MUC1 antigen (**G**) and 60-mer peptide (**H**). The proteolytically derived Fab gave similar binding curves (data not shown). Derived constants for all species are shown in Table 1.

A more detailed analysis of the binding kinetics of the HuHMFG-1 Fab and parent IgG was obtained using SPR with the BIACore 3000. The unmodified scFv described in this study was not analysed as it is an unlikely candidate for further development due to the afore-mentioned solubility and stability limitations during recombinant production. BIACore SPR curves are shown for the whole antibody against a GST-MUC1 fusion protein (Figure 6A and B) and a MUC1 peptide with three tandem repeats (Figure 6E and F). Both showed similar shaped curves with association rates (*k*_a_) values ranging between 1.76 × 10^5^ and 4.2 × 10^5^ M^−1^ s^−1^ and dissociation rates (*k*_d_) values ranging from 0.022 and 0.095 s^−1^ depending on the curve fitting model used (Table 1). As the parent IgG is bivalent a 1 : 1 binding model gave a poor fit and therefore resulted only in an approximation of the kinetic constants. A mass transfer correction did not improve the fit (data not shown). The bivalent model of binding was, as expected, more appropriate for the analysis of the IgG kinetics as indicated by the lower *χ*^2^ value and the derived *k*_a_1 and *k*_d_1 values compare well to those derived from the 1 : 1 fit (Table 1). A steady-state approximation of the *K*_D_ value for the GST-MUC1 (Figure 6C) and 60-mer peptide (Figure 6D) also agrees well with the estimates from the other models. The *K*_D_s ranged from 1.01 × 10^−7^ to 4.9 × 10^−7^ M (Table 1).

The recombinant Fab fragment gave binding curves indicative of lower association and dissociation rates compared to the parent IgG with *k*_a_ values of 865 and 5650 M^−1^ s^−1^ and *k*_d_ values of 0.031 and 0.058 s^−1^ respectively for the GST-MUC1 fusion protein (Figure 6G and the 60-mer tandem repeat (Figure 6F) (Table 1). For comparison, the proteolytically derived HuHMFG-1 Fab gave very similar kinetic values to the recombinant molecule (Table 1).

### Confocal microscopy of HuHMFG-1 internalisation

**Figure 7:**
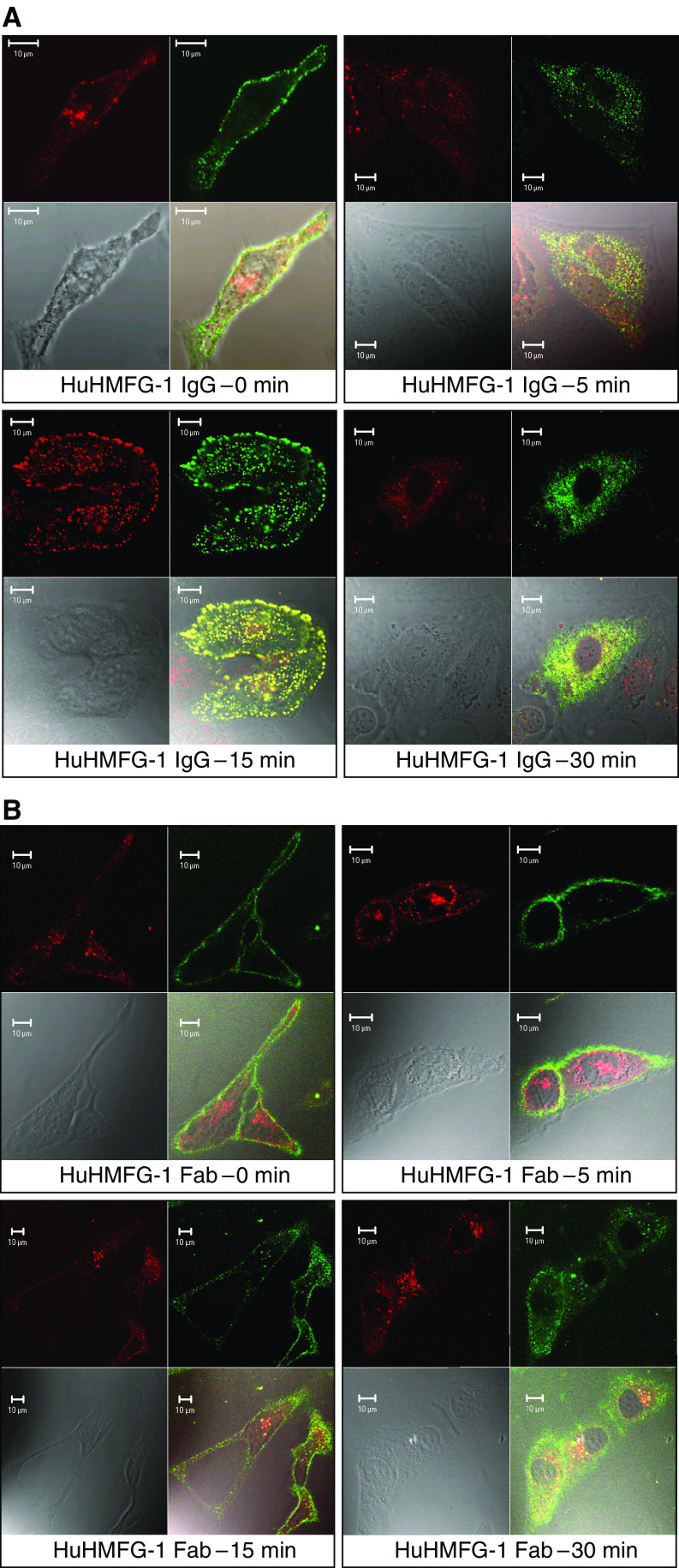
Confocal immunofluorescent binding of HuHMFG-1 (**A**) IgG and (**B**) Fab to MCF-7 breast cancer cells. (**A**) Time course of binding for the IgG detected with anti-Fab FITC (green – upper right panel of each time point). The endosomal compartment of the same cells is visualised with transferrin-Alexa Fluor 594 (red – upper left panel of each time point). The transmission image is shown for each cell (lower left panel of each time point) and the superimposed image is shown for each time point (lower right panel for each time point). (**B**) The same experiment represented the same way for the Fab. The Zeiss LSM 510 software calculates fluorescence colocalisation with a coefficient of 1 meaning completely overlapping signals. For the green and red signals, these values are seen to increase with time, peaking at 15 min where the colocalisation is maximal. Higher colocalisation is seen for the whole IgG. The values are as follows: IgG: 0 min=0.16, 5 min=0.34, 15 min=0.79, 30 min=0.55. Fab: 0 min=0.12, 5 min=0.22, 15 min=0.42, 30 min=0.23).

The internalisation and intracellular trafficking of the HuHMFG-1 antibody/MUC1 complex was analysed by confocal immunofluorescence microscopy. Initially, data from a pilot time course experiment using the HuHMFG-1 IgG were used to select a window within which the relevant intracellular trafficking data could be acquired. Time points of 0, 5, 15 and 30 min were subsequently selected for further analysis. The majority of the HuHMFG-1 IgG was internalised rapidly within 15 to 30 min (Figure 7A), the intracellular staining was punctate and characteristic of early endosomal localisation. This was confirmed with additional colocalisation studies following the internalisation of Alexa Fluor-594 labelled transferrin via the transferrin receptor, which is known to cycle through early endosomes. Significant colocalisation with the labelled transferrin was observed with both the HuHMFG-1 IgG and Fab indicating a similar subcellular trafficking pathway (Figure 7B). The lower affinity of the Fab or its reduced valency, however, resulted in less endosomal localisation compared to the IgG. Coefficients (Figure 7, legend) for transferrin and antibody colocalisation were derived from the immunofluorescence data, showing high correlation with the whole IgG, peaking at 15 min. This peak time of colocalisation is also observed with the Fab fragment but with a lower absolute correlation value. Studies with markers for the endoplasmic reticulum and lysozomes showed no significant colocalisation (data not shown) and no binding or internalisation was seen with the COS-7 MUC1 negative cell line (data not shown). A clearer visualisation of the internalised HuHMFG-1 IgG colocalising with the endosomal marker can be seen in the gallery of images, which shows the cells after 15 min (maximal colocalisation) sliced through the Z-plane (supplementary Figure 1). These images were reconstructed into a three-dimensional model, which is shown rotating 360° in a Quicktime™ movie (supplementary Figure 2). The cells are seen as flattened onto the slide surface, but the depth and internal features can be seen.

## DISCUSSION

This study described the construction and characterisation of the monovalent scFv and Fab fragments of the anti-MUC1 human immunoglobulin HuHMFG-1. Smaller antibody fragments are attractive due to potentially improved tumour penetration and rapid pharmacokinetic properties compared with the IgG format and also offer greater scope and facility for the production of recombinantly derived fusion proteins. Although antigen specificity is generally retained following this type of reformatting the loss of bivalency and the absence of the Fc domain necessitates a detailed *in vitro* analysis prior to any *in vivo* experiments. The analysis of both the HuHMFG-1 scFv and Fab described here benefited from direct comparison with the whole immunoglobulin, which has already undergone extensive clinical testing (Epenetos *et al*, 1982; Seiden and Benigno, 2004).

The poor solubility and stability of the HuHMFG-1 scFv are features that have been commonly observed with other hybridoma-derived scFvs, and further studies have shown that a specific mutation is required to resolve these shortcomings (Krauss *et al*, 2004). Despite this, work here and previous research (Deonarain *et al*, 1997) has shown that sufficient yields can be obtained from refolding inclusion bodies. The improved solubility and stability of the HuHMFG-1 Fab in comparison with the scFv (thereby removing the need for refolding) was therefore predominantly attributed to the presence of the constant domains. Fab fragments often demonstrate preferred *in vivo* pharmacokinetics when directly compared with scFvs as a result of the increase in molecular weight, with many reports describing the suitability of this antibody format for therapeutic applications such as radio-immunotherapy (Alberici *et al*, 1988) or drug delivery (Casey *et al*, 2002).

Previous studies using Scatchard analysis determined the equilibrium dissociation constants (*K*_D_) of the proteolytically derived human Fab fragment as 2.7 × 10^−7^ M respectively (Davies *et al*, 1994). We were unable to accurately determine the affinities of the Fab or scFv by ELISA, but the BIACore SPR analysis gave significantly lower affinity values. It has been observed by others that the HuHMFG-1 IgG has a low affinity for artificial peptide antigens and values for the IgG have been determined to be about 1 × 10^−7^ M (Karanikas *et al*, 1998) agreeing with our observations. The better *K*_D_ values for the antibody species on fixed cells suggests that the antigen, when expressed on the cell surface, exists in a more favourable conformation than that of the GST-MUC1 fusion protein or 60-mer peptide absorbed to a plastic surface or immobilised onto a Carboxy-Methyl dextran chip. The *K*_D_ values for monomeric species of HuHMFG-1 on MCF-7 cells was 33–36 nM, three-fold better than the 113 nM observed for the equivalent scFv in the Krauss *et al* (2004) study. This could be accounted for by differences the level or quality of tumour-associated MUC1 expression on the cells used but supports the relevance of native antigen. The likelihood of crosslinking of the bivalent IgG is potentially increased due to the high-density antigen expression and the multivalent tandem repeat structure of native MUC1. Also, the proposed ‘fist-like’ projection of the tandem repeat loop, which is probably only apparent with cell-tethered MUC1 may promote a higher affinity interaction with the antibody. The HuHMFG-1 antibody was originally raised against native tumour-associated MUC1 and not an artificial peptide and the subtle difference in specificity and affinity is apparent here. Binding analysis on recombinant but similarly glycosylated tumour-associated MUC1 (Backstrom *et al* 2003) would shed more light on this problem. By BIACore SPR (below), the affinity values obtained for the recombinant Fab was approximately 50- to 300-fold lower than the human IgG mirroring the trend of the ELISAs.

Live cells FACS analyses showed that the various HuHMFG-1 species were specific for MUC1 antigen-expressing cells. The fluorescence intensity shifts were similar for the monovalent fragments with the whole IgG showing an almost 10-fold further shift in fluorescence, supporting the cell ELISA observations.

The SPR studies gave a more detailed insight into the binding kinetics of these antibodies with the IgG unusually benefiting from an enhanced association rate compared to the Fab, but with both formats exhibiting similar dissociation rates. It is normally expected that a bivalent antibody species will exhibit a higher overall affinity value due to a slower dissociation rate in comparison with a monovalent species (Nielsen *et al*, 2000). Although the kinetic data for the IgG resulted in a poor fit when analysed using the 1 : 1 interaction model, the *K*_D_ value (1.18 × 10^−7^ M) was similar to that obtained from the bivalent analyte model (4.9 × 10^−7^ M), which gave a closer fit, and also steady-state analysis (1.3 × 10^−7^ M). These results, obtained using the GST-MUC1 fusion protein, were similar to those using the 60-mer MUC1 peptide comprised of three tandem repeats. As expected, the kinetic analysis of the monovalent Fab resulted in a good fit using the standard 1 : 1 model on both the GST-MUC1 and the 60-mer MUC1 peptide with an affinity value approximately 50—to 300-fold lower than that of the IgG. There was practically no difference between the proteolytically derived and recombinant Fab in terms of kinetic binding constants. Compared to other well-characterised antibodies the calculated dissociation rate constants of both the HuHMFG-1 Fab and IgG are rapid (Price *et al*, 1998; Nielsen *et al*, 2000). This was measured on synthetic antigen and could be a major limitation as the overall affinity for native antigen was significantly higher. Despite this, it is likely that antibody dissociation rate is not such a critical factor when targeting rapidly internalised antigens such as MUC1. This is supported by good IC_50_s for tumour cell killing with ribonuclease-based immunofusions *in vitro* (Krauss *et al*, 2004) and immuno-conjugates *in vivo* (Courtenay-Luck *et al*, 2003). The limitations of using synthetic antigens are apparent here and consistent with past observations: the Campath-1H monoclonal antibody (Alemtuzumab), now approved for the treatment of chronic lymphocytic leukaemia (Keating *et al*, 2002), did not bind to its synthetic peptide antigen, but did bind to native deglycosylated or proteolysed peptide (Hale, 1995).

Confocal immunofluorescent microscopy was used to characterise the internalisation of both the HuHMFG-1 Fab and IgG antibody–antigen complexes using live MUC1 expressing cells. Previous studies have indicated that the rate of antibody-MUC1 internalisation may be dependent on the specific epitope that the antibody recognises, with an ‘RPAP’-specific antibody internalising more rapidly than an ‘APDTR’-specific antibody (Pietersz *et al*, 1997). However, the results from our fluorescent microscopy show most of the ‘PDTR’-specific HuHMFG-1 antibody rapidly internalises within 15 min, again demonstrating the importance of fine specificity in internalisation. The lower affinity value of the Fab resulted in less total antibody internalisation but at a comparable rate to the IgG. The MUC1 antigen has been reported to recycle at a rate of 0.9%/min (Altschuler *et al*, 2000), which is slower than would allow such rapid HuHMFG-1 accumulation. However, we are measuring HuHMFG-1 localisation and not MUC1 kinetics, and it is possible that antibody binding or crosslinking may increase MUC1 turnover as is sometimes seen for other antigens. Colocalisation studies of these antibodies with additional endosomal staining (Transferrin-Alexa Fluor 594) showed that the HuHMFG-1 IgG trafficks to early endosomes with increasing colocalisation correlation coefficients peaking at 15 min. Colocalisation and internalisation is clearly seen in the Z-plane images and 3D reconstruction in all parts of the cell. At 30 min some of the HuHMFG-1 is clearly lost to degradation leading to a drop in the correlation coefficients. The Fab fragment exhibited an overall lower degree of colocalisation, presumably related to its significantly lower association rate as determined by SPR, but peaked at the same time point as the IgG.

The C-terminal tail of MUC1 has a number of potential endosomal cycling/sorting motifs, based on the ‘YXXPhi’ sequence (reviewed in Bonifacino and Traub, 2003). These motifs are correctly positioned in MUC1 to interact with the AP adaptor proteins in the clatherin complexes (Bonifacino and Traub, 2003). These sequences are ‘YGQL’, ‘YEKV’ and ‘YPTY’, the latter being closest to the consensus. These sequences exhibit similarity with the known motif found in the transferrin receptor ‘YTRF’ (Bonifacino and Traub, 2003). This may explain the similar intracellular localisation patterns observed with the immunofluorescence microscopy. Whereas the transferrin receptor does not play a signalling role upon endosomal cycling, there is accumulating evidence that MUC1 does have some signal transduction function. This is supported by the fact that some of these sorting motifs are within phosphorylated signalling motifs (Zrihan-Licht *et al*, 1994; Wang *et al*, 2003).

In conclusion, we have determined the affinities and kinetic binding constants for monovalent and bivalent human HMFG-1 antibodies and shown how this related to cell binding and route of internalisation. A rapid dissociation rate was determined but this has not adversely affected therapeutic strategies such as targeted apoptosis, where cytotoxic enzymes are delivered to the cytosol of tumour cells.

## Figures and Tables

**Table 1:** Surface plasmon resonance kinetic analyses of antibody binding

	**Gst-MUC1 fusion protein (1-repeat)**			**60-mer MUC1 peptide (3-repeats)**		
	**1 : 1**	**Bivalent**	**Steady state**	**1 : 1**	**Bivalent**	**Steady state**
*HuHMFG-1 IgG*						
*k*_a_ (M^−1^s^−1^)	4.2 × 10^5^	1.76 × 10^5^	NA	4.1 × 10^5^	2 × 10^5^	NA
*k*_a_ (s^−1^)	0.049	0.086	NA	0.022	0.095	NA
*K*_D_ (M)	1.18 × 10^−7^	4.9 × 10^−7^	1.3 × 10^−7^	1.83 × 10^−7^	1.68 × 10^−7^	1.01 × 10^−7^
Fit (*χ*^2^)	30	6	8	50	5	9

*HuHMFG-1 Fab-(recomb.)*						
*k*_a_ (M^−1^s^−1^)	865	NA	ND	5650	NA	ND
*k*_d_ (s^−1^)	0.031	NA	ND	0.058	NA	ND
*K*_D_ (M)	3.6 × 10^−5^	NA	ND	1 × 10^−5^	NA	ND
Fit (*χ*^2^)	17	NA	ND	12	NA	ND

*HuHMFG-1 Fab (prot.)*						
*k*_a_ (M^−1^s^−1^)	1310	NA	ND	5727	NA	ND
*k*_d_ (s^−1^)	0.038	NA	ND	0.063	NA	ND
*K*_D_ (M)	2.9 × 10^−5^	NA	ND	1.1 × 10^−5^	NA	ND
Fit (*χ*^2^)	19	NA	ND	15	NA	ND

Association and dissociation rate constants are shown for the binding of HuHMFG-1 IgG and Fab, on Gst-MUC1 and 60-mer MUC1 peptides. Each binding experiment was fit to a 1 : 1 Langmuir binding model and bivalent analyte model (BIAevaluation 3.01) and the degree of fit is indicated by the *χ*^2^ value. The derived equilibrium dissociation constant is shown as well as a determination by steady-state analysis. NA=not applicable, ND=not determined.
