# Supplementary material for: Characterisation and internalisation of recombinant humanised HMFG-1 antibodies against MUC1
Source: Br J Cancer. 2005 Nov 1;93(11):1257–66. doi: 10.1038/sj.bjc.6602847 (PMC3216111; doi:10.1038/sj.bjc.6602847)
Supplement: Supplementary Figure Legends [file 93-6602847x1.doc]

**Supplementary Figures**

**Figure 1**

**Z-plane images**

Gallery of images through the Z-plane of SKOV-3 cells staining for HuHMFG-1 and transferrin receptor at maximun co-localisation (15 minutes). Twenty image slices were taken moving through the cell.

**Figure 2**

**QuickTime movie of rotating cell**

The images from supplementary Figure 1 were processed using the Volocity software to reconstruct a 3-dimensional image of the cell. This was rotated 360° and captured as a movie which shows internal features and internalisation and co-localisation throughout the cell.
