# Supplementary figures and images for: Characterisation and internalisation of recombinant humanised HMFG-1 antibodies against MUC1
Source: Br J Cancer. 2005 Nov 1;93(11):1257–66. doi: 10.1038/sj.bjc.6602847 (PMC3216111; doi:10.1038/sj.bjc.6602847)

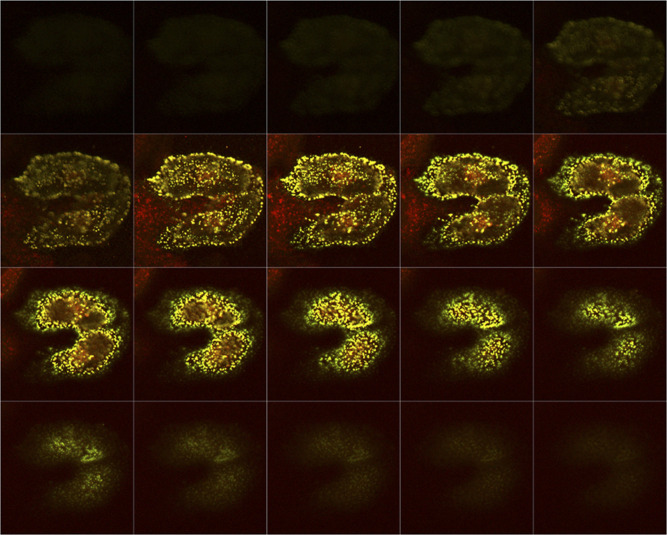

Supplement: Supplementary Figure 1 [file 93-6602847x2.jpg]
